# Reverse Takotsubo Cardiomyopathy After Accidental Exposure to an Illicit Substance: A Case Report

**DOI:** 10.7759/cureus.24282

**Published:** 2022-04-19

**Authors:** Charles White, Rebecca Jeanmonod

**Affiliations:** 1 Emergency Medicine, St. Luke's University Health Network, Bethlehem, USA

**Keywords:** amphetamine toxicity, case report, amphetamine related cardiomyopathy, cardiomyopathy, takotsubo

## Abstract

Stress-induced (Takotsubo) cardiomyopathy is a clinical syndrome and its incidence has been on the rise. Patients with this syndrome often present with chest pain, dyspnea, or syncope. The findings from a typical cardiac evaluation can make this entity difficult to distinguish from acute myocardial infarction (AMI).

A 50-year-old woman presented to the emergency department (ED) with anxiety and palpitations after the accidental ingestion of a sympathomimetic. The patient had consumed coffee that she had brewed in a hotel coffee pot, unaware that a previous guest had placed drug paraphernalia including methamphetamine in the water reservoir of the coffee pot. Her symptoms had started shortly thereafter. In the ED, the patient’s workup was remarkable for positive troponin and an echocardiogram demonstrating basilar hypokinesis. The patient’s ejection fraction was reduced at 40%. She was admitted to the hospital, where she underwent catheterization, demonstrating normal coronary arteries. She had full clinical recovery at the six-month follow-up.

Reverse Takotsubo cardiomyopathy is a rare variant of Takotsubo cardiomyopathy, which presents with basal left ventricular hypokinesis. It is associated with younger age and female gender and has an overall good prognosis. This diagnosis should be considered in patients who are otherwise at low risk for atherosclerotic cardiac disease with known emotional or physical triggers and changes in left ventricular function. Treatment is generally supportive, but a Takotsubo cardiomyopathy diagnosis alters the management of shock and dysrhythmia when known.

## Introduction

Takotsubo cardiomyopathy, or stress-induced cardiomyopathy, is a clinical syndrome that manifests with reversible left ventricular systolic dysfunction without associated coronary artery disease [1,2]. The most common type of Takotsubo cardiomyopathy demonstrates apical hypokinesis of the left ventricle, resulting in the appearance of apical “ballooning” on echocardiographic images [1,2]. There are variant forms of Takotsubo cardiomyopathy that present with different patterns of ventricular systolic dysfunction and have slightly different clinical characteristics as well as prognoses [3]. We present a case of reverse Takotsubo cardiomyopathy that developed after accidental exposure to an illicit substance.

## Case presentation

A 50-year-old woman presented to the emergency department (ED) with palpitations, anxiety, and tingling paresthesias of her lips and extremities. She had been traveling from out of town, and her symptoms had begun shortly after drinking coffee that she had brewed in the hotel room coffee pot. After the symptoms had developed, the patient examined the coffee pot and found drug paraphernalia (glass pipes and an unmarked bag with a crystal-like substance) within the water reservoir. The patient had contacted the authorities and subsequently presented for care. She denied chest pain, dyspnea, abdominal pain, nausea, or vomiting. A review of systems was otherwise negative. She reported no medications and denied any intentional use of illicit substances. She exercised regularly.

On exam, the patient had normal vital signs. In general, she appeared anxious and distraught, with some mild diaphoresis. Her cardiopulmonary and abdominal exams were normal. On neurologic exam, her pupils were dilated, symmetric, and reactive. She had no nystagmus and no clonus or fasciculations. The remainder of her exam was noncontributory.

**Figure 1:**
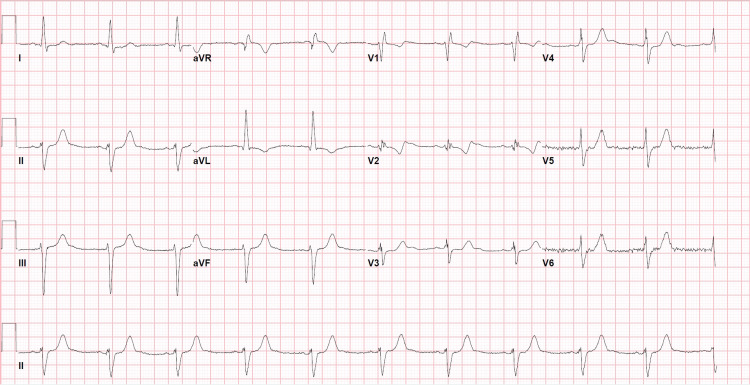
Electrocardiogram demonstrating nonspecific repolarization abnormalities

ECG demonstrated normal sinus rhythm and left ventricular hypertrophy with a widened QRS and a repolarization abnormality (Figure 1).

The patient’s chest X-ray was normal. Her troponin level was elevated at 2.36 ng/ml (reference range: <0.04 ng/ml). The urine drug screen was positive for amphetamines/methamphetamines. The patient underwent an emergent echocardiogram, which demonstrated an ejection fraction of 40% as well as dyskinesis of the basal-mid anteroseptal, basal-mid inferoseptal, and basal-mid inferior walls. The three attached videos demonstrate these findings (Videos 1-3).

**Video 1:** Echocardiogram demonstrating basal-mid anteroseptal, basal-mid inferoseptal, and basal-mid inferior walls

**Video 2:** Ultrasound demonstrating dyskinesis of the basal-mid anteroseptal, basal-mid inferoseptal, and basal-mid inferior walls

**Video 3:** Echocardiogram demonstrating dyskinesis of the basal-mid anteroseptal, basal-mid inferoseptal, and basal-mid inferior walls

Cardiac catheterization revealed normal coronary arteries and confirmed severe basal-mid anteroseptal, basal-mid inferoseptal, and basal-mid inferior wall dyskinesis, consistent with reverse Takotsubo cardiomyopathy (Videos 4-6). The patient was treated with supportive care and discharged on hospital day three. At the six-month follow-up, she had a full clinical recovery.

**Video 4:** Cardiac catheterization demonstrating normal coronary arteries - clip 1

**Video 5:** Cardiac catheterization demonstrating normal coronary arteries - clip 2

**Video 6:** Left ventriculogram demonstrating dyskinesis and reduced ejection fraction of 40%

## Discussion

Reverse Takotsubo cardiomyopathy is a rare variant of Takotsubo cardiomyopathy. Takotsubo cardiomyopathy, also known as stress-induced cardiomyopathy, is an acute, reversible, left ventricular dysfunction that is primarily triggered by physical or emotional stressors, although it has also been reported with no evident trigger [1,2]. In a study of 1,750 patients with Takotsubo cardiomyopathy, physical stressors (36%) were more frequent than emotional stressors (27.7%) [1]. Takotsubo cardiomyopathy is characterized by apical hypokinesis without obstructive coronary disease in 81.7% of patients [1]. Atypical variants of Takotsubo cardiomyopathy have been described, including hypokinesis of the mid ventricle in 14.6%, basal hypokinesis (reverse Takotsubo cardiomyopathy) in 2.2%, and focal hypokinesis in an isolated region of the left ventricle in 1.5% [1].

While the underlying pathophysiology of Takotsubo cardiomyopathy and its variants is unknown, it is thought that sympathetic hyperactivity plays a pivotal role, resulting in vasospasm, microvascular dysfunction, and direct cardiotoxicity [3,4]. A recent Japanese study described an increased rate of Takotsubo cardiomyopathy among patients on prescribed intravenous adrenergic agents, and case reports have shown an association with neurogenic causes as well as amphetamine use [5-7]. Animal model research links circulating microRNAs associated with anxiety and depression to reduced myocardial contractility when exposed to adrenergic agents, suggesting that these may be predisposing factors in Takotsubo cardiomyopathy development [8].

Reverse Takotsubo cardiomyopathy in particular appears to be linked with amphetamine use and the female gender [6,9]. Patients with reverse Takotsubo cardiomyopathy are also typically younger, with a mean age of 36 years (as compared to 62 years in Takotsubo cardiomyopathy) [10]. Although other forms of Takotsubo cardiomyopathy may have no clear triggers, reverse Takotsubo cardiomyopathy has almost exclusively been found in patients with a clear precipitating stressor, including pheochromocytoma, amphetamine use, eating disorders, and attempted suicide by hanging [11,12].

The most common presenting symptoms of Takotsubo cardiomyopathy are chest pain, dyspnea, and syncope [1,4,13]. Patients may also present with acute congestive heart failure, cardiogenic shock, or dysrhythmias [4]. ECG findings include ST-segment elevation (particularly in aVR), ST depressions, left bundle branch block, prolonged QT, and T wave inversion [4]. These findings are nonspecific, making differentiation between acute myocardial infarction (AMI) and Takotsubo cardiomyopathy challenging. Additionally, both conditions can elevate troponin levels. Echocardiography can distinguish between AMI and Takotsubo cardiomyopathy if the classic findings of Takotsubo cardiomyopathy or reverse Takotsubo cardiomyopathy are present; however, cardiac catheterization is the gold standard method to exclude AMI.

Acute management of patients with Takotsubo cardiomyopathy is generally supportive in nature. There is little guidance on how to manage the 6-20% of patients who develop cardiogenic shock [2]. Intuitively, based on pathophysiology, adrenergic agents should be avoided, as they may worsen or prolong the condition [2]. Beta-blockade has not been associated with improved outcomes in non-randomized studies, and mechanical circulatory support, used to bridge patients to spontaneous recovery, remains unstudied [2]. The most common acute arrhythmias in Takotsubo cardiomyopathy are ventricular dysrhythmias and complete heart block [2]. If Takotsubo cardiomyopathy is known or highly suspected, beta-blockade and magnesium are appropriate initial therapies for ventricular dysrhythmias that do not require cardioversion [2]. Because of the high incidence of QTc prolongation in these patients, amiodarone should be withheld until screening ECG is obtained [2].

Most patients with Takotsubo cardiomyopathy recover within six months. However, 7.1% of patients experience major adverse cardiac and cerebrovascular events in the first 30 days of diagnosis, with worse outcomes in patients with neurologic precipitant of Takotsubo cardiomyopathy, cardiogenic shock during hospitalization, older age, or significant comorbidities [2]. Mitral regurgitation and right ventricular involvement are independently associated with poorer prognosis [2]. Patients with an emotional trigger for Takotsubo cardiomyopathy, younger patients, and females tend to do well [2].

The etiology of this case of reverse Takotsubo cardiomyopathy may have been multifactorial. The amphetamines/methamphetamines the patient accidentally ingested may have been a direct cause of reverse Takotsubo cardiomyopathy, as these agents have been linked to reverse Takotsubo cardiomyopathy [14]. The patient’s intense emotional response upon discovering the drug paraphernalia within the coffee pot water reservoir may also have precipitated reverse Takotsubo cardiomyopathy. The patient was very anxious during hospitalization, requiring several doses of anxiolytic medication before she could tolerate echocardiogram and cardiac catheterization, and anxiety disorders have been implicated as one of the most common predisposing factors in Takotsubo cardiomyopathy [15]. Hence, in this case, both etiologies may have played a role in the pathophysiology of the patient’s disease.

## Conclusions

Reverse Takotsubo cardiomyopathy is a rare variant of Takotsubo cardiomyopathy, which presents with basal left ventricular hypokinesis. It is associated with younger age and female gender and has an overall good prognosis. This diagnosis should be considered in patients who are otherwise at low risk for atherosclerotic cardiac disease with known emotional or physical triggers and changes in left ventricular function. A bedside echocardiogram can be helpful in diagnosis, although ultimately, a definitive diagnosis of Takotsubo cardiomyopathy requires a negative cardiac catheterization. Treatment is generally supportive, but if Takotsubo cardiomyopathy is suspected, it is reasonable to avoid adrenergic agents and amiodarone.
